# Rational selection and engineering of exogenous principal sigma factor (σ^HrdB^) to increase teicoplanin production in an industrial strain of *Actinoplanes teichomyceticus*

**DOI:** 10.1186/1475-2859-13-10

**Published:** 2014-01-16

**Authors:** Haiyong Wang, Liu Yang, Kuo Wu, Guanghui Li

**Affiliations:** 1 School of Food and Bioengineering, Qilu University of Technology, Jinan 250353, PR China; 2 Biotechnology and Genetic Resources institute, Yunnan Agricultural Academy, Kunming 650223, PR China; 3 Department of Chemistry, Tangshan Normal University, Tangshan 063000, PR China

**Keywords:** *Actinoplanes teichomyceticus*, Teicoplanin, Metabolic perturbation, Exogenous principal sigma factor

## Abstract

**Background:**

Transcriptional engineering has presented a strong ability of phenotypic improvement in microorganisms. However, it could not be directly applied to *Actinoplanes teichomyceticus* L-27 because of the paucity of endogenous transcription factors in the strain. In this study, exogenous transcription factors were rationally selected and transcriptional engineering was carried out to increase the productivity of teicoplanin in L-27.

**Results:**

It was illuminated that the σ^HrdB^ molecules shared strong similarity of amino acid sequences among some genera of actinomycetes. Combining this advantage with the ability of transcriptional engineering, exogenous sigma factor σ^HrdB^ molecules were rationally selected and engineered to improve L-27. *hrdB* genes from *Actinoplanes missouriensis* 431, *Micromonospora aurantiaca* ATCC 27029 and *Salinispora arenicola* CNS-205 were selected based on molecular evolutionary analysis. Random mutagenesis, DNA shuffling and point mutation were subsequently performed to generate diversified mutants. A recombinant was identified through screening program, yielding 5.3 mg/ml of teicoplanin, over 2-fold compared to that of L-27. More significantly, the engineered strain presented a good performance in 500-l pilot scale fermentation, which meant its valuable potential application in industry.

**Conclusions:**

Through rational selection and engineering of exogenous transcriptional factor, we have extended the application of transcriptional engineering. To our knowledge, it is the first time to focus on the related issue. In addition, possessing the advantage of efficient metabolic perturbation in transcription level, this strategy could be useful in analyzing metabolic and physiological mechanisms of strains, especially those with the only information on taxonomy.

## Introduction

Many microorganisms have been developed as cell factories to produce bioactive chemicals. The growing commercial application urges us to seek more effective approaches for production improvement of these chemicals. Many efforts have been focused on strain improvement and its bioprocess control. Several approaches to bioprocess control have been explored, modulating the culture environment, optimizing media components and sharpening downstream bioprocess included. While its productivity is mainly determined by the native metabolic properties of the microorganism under a stable industrial condition, strain improvement has attracted more attentions. In general, three approaches have been employed in strain improvement: i) Random mutation at the cellular level, which is labor and time intensive in spite of successful application in production units; ii) Rational metabolic engineering at the molecular level, which is limited by detailed profiles of metabolic pathways; and iii) Semi-rational metabolic engineering that mimics the natural evolutionary process, which often involves biological principle-based design and pre-random mutagenesis at the molecular or cellular level. Given that perturbation of the whole metabolic network can efficiently produce significant genetic diversity, the third approach has recently been prevalent.

The illumination of transcriptional mechanisms in microorganisms [1,2] has promoted the application of transcriptional engineering strategy. In the strategy, free of hazardous reagents involved, intensive labor and consuming time, both cis-acting elements [3,4] and trans-acting factors have been engineered [5-8] to direct the metabolic flux in a desired way through random mutagenesis and screening. As a typical trans-acting factor, principal sigma factor can be engineered to rebuild the whole-cell metabolism for the rapid optimization of strain phenotypes. The transcriptional engineering has been successfully applied in yeast [9], *Escherichia coli*[10] and *Lactobacillus plantarum*[11]. Considering its strong capability of regulating diverse metabolic and physiological functions, this approach is also urgently used to engineer more strains, especially the industrial valuable and newly explored ones.

As was known, teicoplanin played a key role in the treatment of serious infections caused by drug-resistant bacteria [12] and chronic hepatitis C [13]. We recently aimed at its industrial producer strain *A. teichomyceticus* L-27. For practical application, the bioprocess of *A. teichomyceticus* has been previously determined [14]. In addition, tapping the potential yield, several rounds of strain engineering were operated, including random mutation [15] and limited metabolic engineering [16]. By this way, there was less margin remained for further phenotypic improvement in *A. teichomyceticus* L-27. Therefore, exogenous principal sigma factor σ^HrdB^ (coded by *hrdB* gene), a kind of global transcription factors, was selected and engineered through transcriptional engineering to refine the valuable cell factory. Our approach has extended the application of transcriptional engineering and is an effective alternative to random mutation for some industrial and newly strains with poor metabolic information.

## Methods

### Strains and plasmids

The bacterial strains and plasmids used in this study were preserved in our lab. *E. coli* DH5α and *E. coli* ET12567/pUZ8002 were used for convenient cloning, propagating and conjugating DNA molecules into *A. teichomyceticus*. Plasmids pUC19-AmhrdB, pUC19-MahrdB and pUC19-SahrdB harbored the synthesized *hrdB* coding sequences from *Actinoplanes missouriensis* 431, *Micromonospora aurantiaca* ATCC 27029 and *Salinispora arenicola* CNS-205, respectively. These *hrdB* fragments were flanked by a pairs of adaptors (5′CTCGCCTGCGGGACAGG*ACTAGT*3′ for upstream, the *Spe*I restriction site was italicized; 5′GGTGAGCAGACCG*GAATTC*CG3′ for downstream, the *Eco*RI restriction site was italicized). An integration vector pGH112-PR, a derivative of the plasmid pGH112 [17], harbored ermE promoter, ribosome binding and *Spe*I-*Bam*HI-*Nhe*I-*Eco*RI multiple cloning sites. It was used for dominant phenotype screening of thiostrepton and ampicillin resistance in actinomycetes and *E. coli*, respectively.

### Culture methods

An industrial strain *A. teichomyceticus* L-27 was grown in the medium of glucose (10.00 g/l), yeast extract (2.50 g/l), K_2_HPO_4_ (1.00 g/l), KCl (5.00 g/l), MgSO_4_ · 7H_2_O (0.50 g/l), FeSO_4_ (0.01 g/l) and agar (20.00 g/l, added for solid medium), in which the vegetative mycelium propagated. *E. coli* DH5α was grown in Luria–Bertani (LB) medium, while *E. coli* ET12567 (pUZ8002) harboring the donor plasmid was propagated in LB medium with appropriate antibiotics.

### Teicolplanin fermentation program

Seed medium was made by adding corn flour (40.00 g/l), glucose (10.00 g/l), soybean flour (2.50 g/l), peptone (2.50 g/l), yeast extract (2.50 g/l), (NH_4_)_2_SO_4_ (2.50 g/l), NaCl (4.00 g/l), MgSO_4_ · 7H_2_O (0.25 g/l) and CaCO_3_ (4.00 g/l). The fermentation medium contained corn flour (40.00 g/l), glucose (20.00 g/l), soybean flour (6.00 g/l), peptone (2.00 g/l), yeast extract (2.50 g/l), MgSO_4_ · 7H_2_O (0.30 g/l) and CaCO_3_ (3.00 g/l). Solid cultivation was performed in a culture box at 28°C, liquid cultivation at 29°C on a rotator shaker (220 rpm). Valine (4 mg/l), arginine (4 mg/l) and n-butanol (4 mg/l) were added into the culture at the appropriate fermentation time of 60 h, 60 h and 30 h, respectively. The total fermentation time was 120-140 h.

### Analytical method

The fermentation broth was adjusted to pH 10.0-11.5 and stirred for 2 h at ≤ 20°C. The resultant broth was filtrated and diluted to an appropriate concentration for high performance liquid chromatography (HPLC) analysis. HPLC (Lab-Alliance, USA) was used for determining the quality and quantity of teicoplanin. Column: Packing with Comatex C18, 5 μm; Size: 250 × 4.6 mm, connected to guard column C18; Mobile phase: A 0.02% ammonium acetate/acetonitrile = 95/5, B 0.02% ammonium acetate/acetonitrile = 25/75; The initial conditions were 32% of eluent B with linear gradient to 50% in 10 min at flow rate of 1 ml/min; UV detector wavelength: 277 nm.

### Molecular evolutionary analysis

For phylogenetic analyses, the amino acid sequences of principal sigma factor σ^HrdB^ were aligned using Clustal X 1.83 [18]. Phylogenetic trees were constructed with the neighbor-joining statistical method of the p-distance model using the MEGA5.2 program [19]. The reliability of the trees was computed by 1000 replications of bootstrap analyses.

### Directed evolution of σ^HrdB^

Both error-prone PCR and DNA shuffling were used here to evolve *hrdB* in vitro. Three donor *hrdB* fragments were amplified from the templates pUC19-AmhrdB, pUC19-MahrdB and pUC19-SahrdB using universal primer pairs (H-F/H-R: 5′CTCGCCTGCGGGACAGG*ACTAGT*3′, the *Spe*I restriction site was italicized; 5′CG*GAATTC*CGGTCTGCTCACC3′, the *Eco*RI restriction site was italicized). They were then purified from using SanPrep gel extraction kit (Sangon, Shanghai, China). 4 ng of reclaimed products was used as templates in the StEP [20] system, which contained 3.0 U Taq DNA polymerase, 10% DMSO, 4 × 200 μM dNTP and 400 nM primer pairs H-F/H-R. The program was designed to cycle 99 times at 95°C for 30 s followed by 55°C for 5 s. The rescued *hrdB* fragment was randomly mutated by error-prone PCR with primer pairs H-F/H-R using GeneMorph^®^ II Random Mutagenesis Kit (Stratagene, La Jolla, USA) according to the manufacturer’s instructions. The products were recovered by SanPrep gel extraction kit. Mutated products derived from both error-prone PCR and shuffling process were digested by *Spe*I and *EcoR*I, inserted into pGH112-PR, and transformed into competent *E. coli* ET12567/pUZ8002 to generate *hrdB* mutant library.

### Mutant screening and identification program

The resulting *hrdB* mutant library was transferred into *A. teichomyceticus* L-27 following standard procedures [21]. The high-throughput screening for high-yield strains was performed as described previously [22] with some modifications. The solid-state 96 deep well micropaltes were incubated for 8 days at 28°C and then extracted with 1 ml 50% ethanol for 4 h. The 50 μl supernatant was mixed with 200 μl 50% ethanol in a new plate, and the UV absorbance was measured at 277 nm.

The evolved *hrdB* integrated into the genomic DNA of the selected recombinants were cloned by PCR using primer pairs P-F/A-R (5'CACCGCGACGCTGTTGTG3′, 5′CGGAATTCCGGTCTGCTCACC3′), sequenced and aligned with their parent using Clustal X 1.83. PCR-amplified *hrdB* fragments were cloned into pGH112-PR and then transformed into the strain *A. teichomycetic*us L-27, expelling false positive. The desired clone was further identified by the second round of fermentation test. The importance and necessity of point mutations created through error-prone PCR for improved host was confirmed by site-directed mutation. Site-directed mutagenesis was performed using the stratagene quickchange kit (Stratagene, La Jolla, USA). The mutation was introduced into the exogenous *hrdB* gene from P-22 with primer pairs: h142-F/h142-R (5'CGAAGACCGCAGCGG*T*CAAGCCGGCCAA GGC3′, 5'GCCTTGGCCGGCTTG*A*CCGCTGCGGTCTTCG3′) and h354-F/h354-R (5'CACCCGCG CCATGGCC*C*ACCAGGCCCGCACCATC3′, 5'GATGGTGCGGGCCTGGT*G*GGCCATGGCGCGG GTG3′).

## Results

### Molecular evolutionary analysis of σ^HrdB^

**Figure 1:**
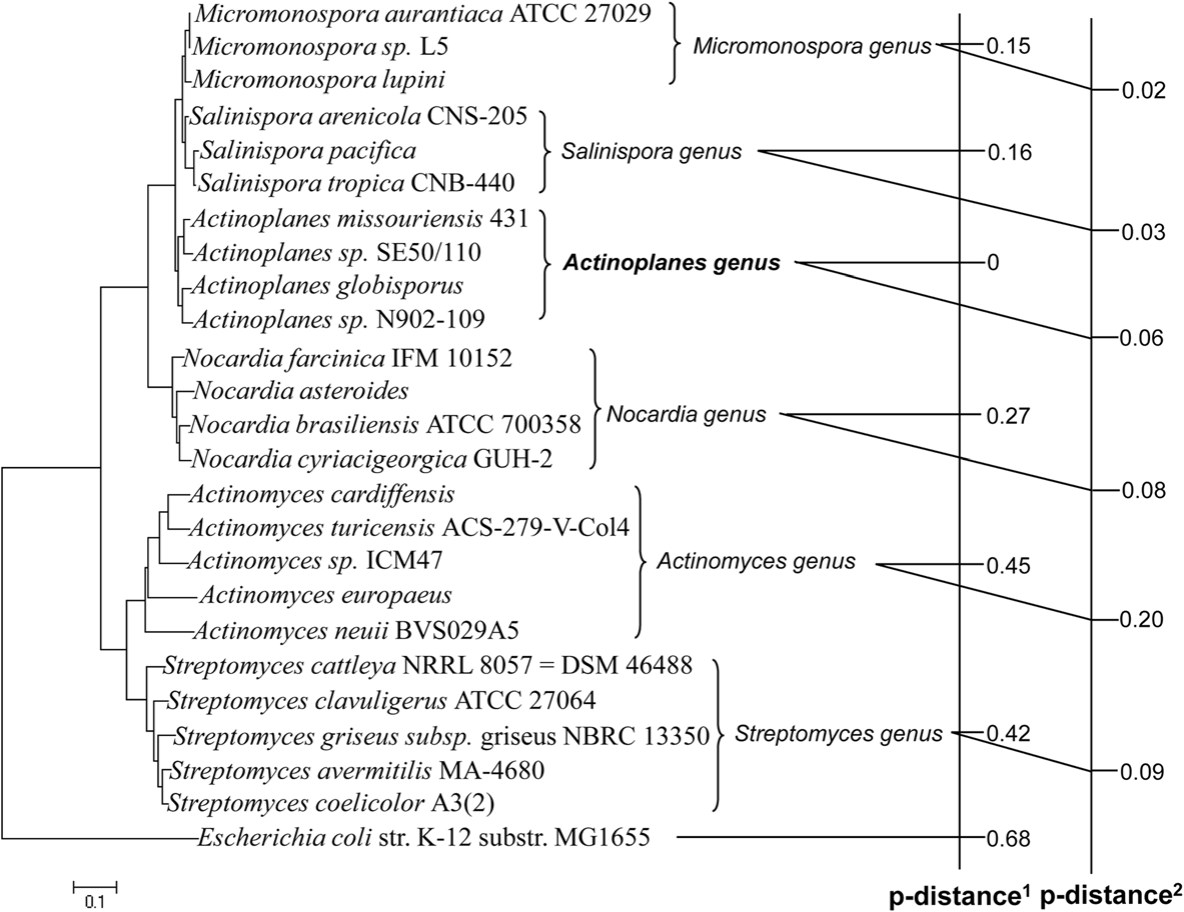
**Neighbor-joining phylogenetic tree of the σ**^**HrdB **^**molecules.** With bootstrap value of 1000 replicates, phylogenetic tree of the σ^HrdB^ molecules was constructed. The p-distance^1^ depicted here was derived from running program ‘compute between group mean distance’ using software MEGA5.2 (standard error less than 0.02). Average within-group p-distance^2^ of each genus was 0.08 (standard error = 0.01) when the program ‘compute within group mean distance’ was run.

The nucleic acid sequence of native *hrdB* in *A. teichomyceticus* was unidentified and remained a gap in the application of transcriptional engineering. As an alternative, exogenous *hrdB* should be rationally selected. Since *A. teichomyceticus* L-27 possessed the taxonomic status of *Actinoplanes* genus in actinomycetes, the neighbor-joining phylogenetic tree of several strains in typical actinomycete genera was constructed based on amino acid sequences of σ^HrdB^. Among the 25 σ^HrdB^ molecules (or their homologue), 24 derived from actinomycetes were regularly clustered in six genera of actinomyctes (Figure 1).

Although there were high similarities of several conservative regions among principal sigma factors from diverse organisms [23], significant changes in amino acid residues occurred in the different genera of actinomycetes, presented by average p-distance 0.15-0.45 between *Actinoplanes* and other five groups. Besides, the average p-distance was up to 0.68 using a distant organism *Escherichia coli str. K-12 substr.* MG1655 as a reference. The phylogenetic tree also showed that three genera *Actinoplanes*, *Micromonospora* and *Salinispora* were made up of a clade, where the p-distances were no more than 0.16. Smaller within-group p-distance 0.02-0.2 was also acquired, which meant that there was no apparent evolutionary divergence in σ^HrdB^ among each genus of actinomycetes. In view of possibility of mutational robustness disruption of σ^HrdB^ molecules, several relatives of L-27 from the above three genera were selected as donors of exogenous *hrdB* to patch the gap. The exogenous global transcription factor σ^HrdB^ was engineered to improve the desired phenotype in L-27.

### *hrdB* mutant library construction and screening

**Figure 2:**
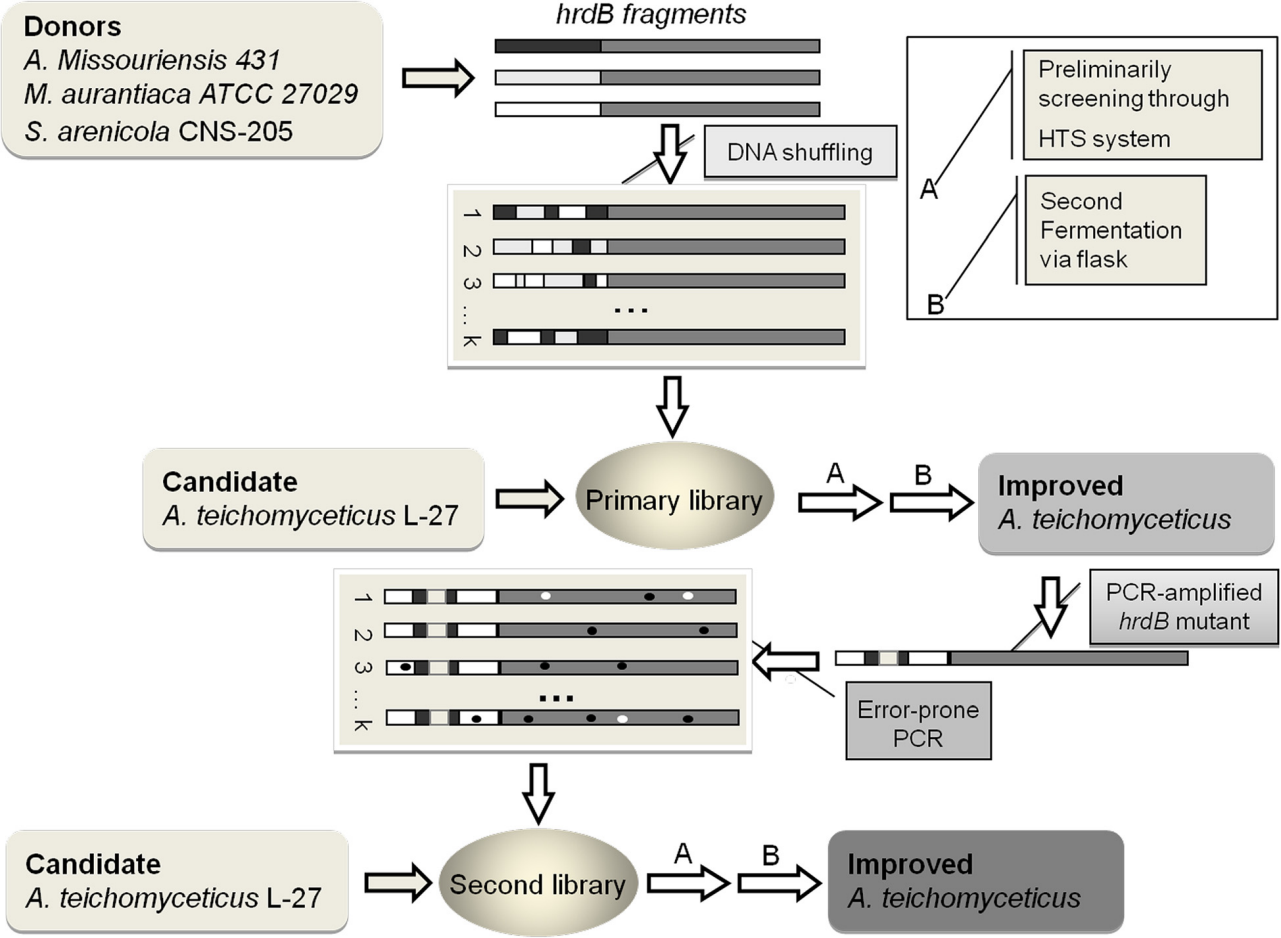
**Schematic representation of exogenous transcription factor engineering.** Improved recombinants would be screened and selected from both two mutant libraries (primary library and second library).

To diversify and redirect the metabolic flux to the teicoplanin biosynthesis through transcriptional regulation, exogenous *hrdB* random mutant libraries were constructed and screened (Figure 2). The *hrdB* fragments respectively derived from *Actinoplanes missouriensis* 431, *Micromonospora aurantiaca* ATCC 27029 and *Salinispora arenicola* CNS-205, as original genetic pool, were firstly subjected into StEP system to generate a primary library containing approximately 1 × 10^5^ clones. The preliminary screening process was performed in high throughput screening system. About 700 clones were screened, of which 27 were subjected to the second round of screening in flask fermentation and HPLC analysis. To expel false positive clones, the PCR-amplified mutant *hrdB* fragments were rescued and then transferred into L-27. Among these strains, the best recombinant named P-22 was identified. Subsequently, the shuffled *hrdB* fragment from P-22 was mutated using error-prone PCR at mutation frequency of 4-9 mutations/kb, and the second library harboring about 6.4 × 10^5^ clones was generated. 53 strains were evaluated in flask cultures to determine the productivity after preliminary screening process.

**Figure 3:**
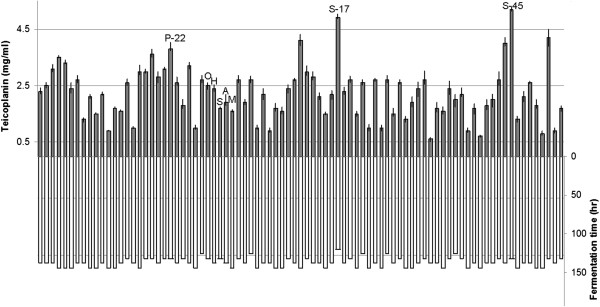
**Screening of teicoplanin overproduction recombinants harboring evolved mutant *hrdB *gene cultured in flasks.** All samples were measured in duplicate. Error bars indicated standard deviations. The reference strains were O (L-27), H (L-27∷pGH112-PR), A (L-27∷*AmhrdB*), M (L-27∷*MahrdB*) and S (L-27∷*SahrdB*).

The total of 85 strains (5 reference strains contained) showed different fermentation performance, in which P-22 produced 3.8 mg/ml of teicoplanin, over 1.5-fold compared to that of the original strain (Figure 3). On the other hand, two other strains S-17 and S-45 were selected from the second library. S-17 with a shortened fermentation period (120 h) possessed the second highest yield of 4.9 mg/ml of teicoplanin. The best recombinant S-45 was confirmed in this work, producing 5.3 mg/ml of teicoplanin.

### Profiles of S-17 and S-45 in pilot-scale fermentation

**Figure 4:**
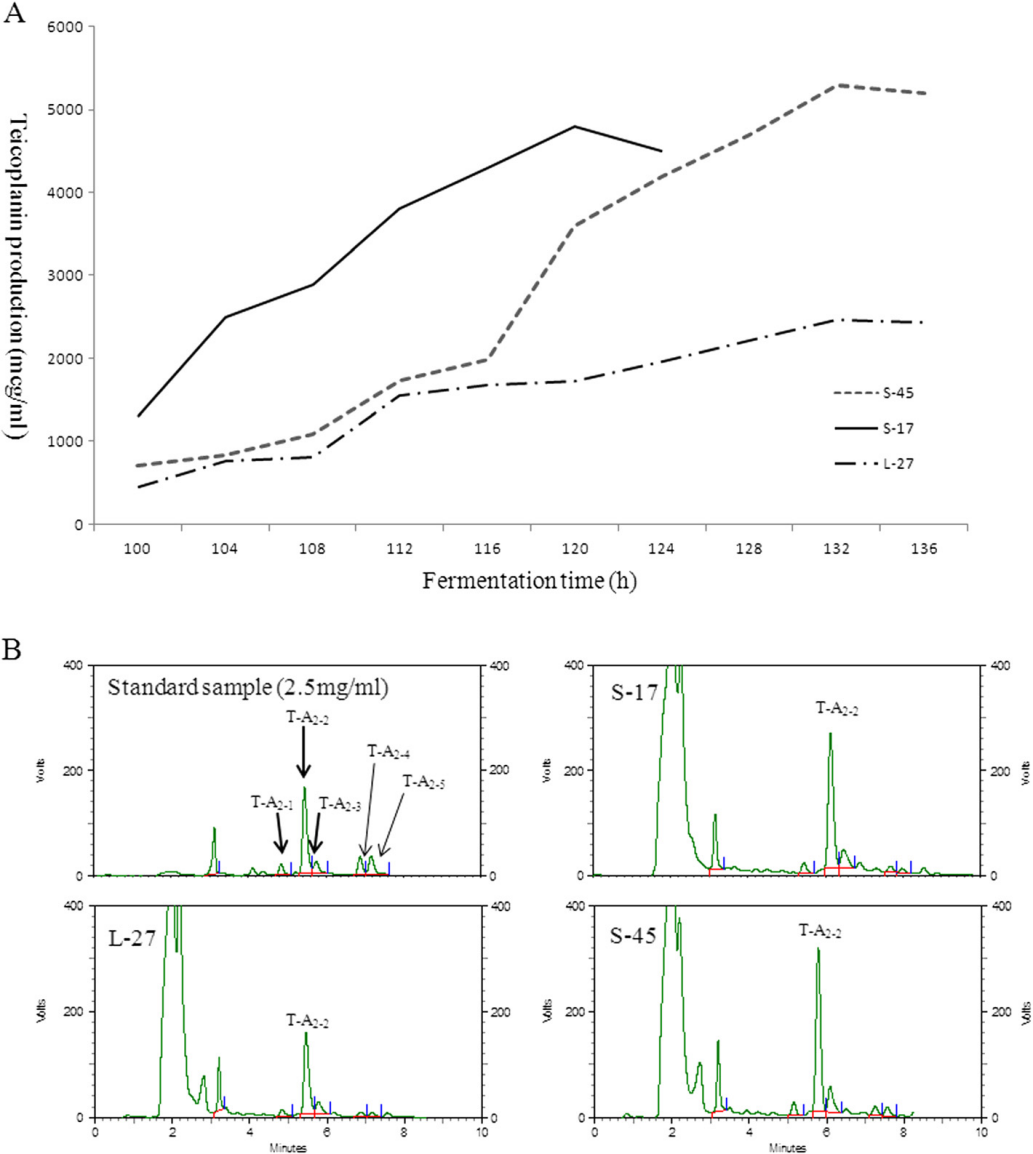
**The 500 l pilot-scale fermentation performances of the parental strain L-27 and the resulting strains S-17 and S-45.** The fermentation performance was defined by the fermentation time and teicoplanin yield of the recombinants compared to that in L-27 in this study **(A)**. HPLC profiles of teicoplanin extracts were also shown **(B)**. All samples were diluted 1:5 with water.

To further assess the reproducibility in pilot scale and the effectiveness of microbial engineering, S-17 and S-45 were cultivated in a 500 l fermentor. As shown in Figure 4, S-45 fermentation resulted in an increase of 100% of the teicoplanin yield, more than that in the parent strain. Simultaneously, S-17 exhibited comparable teicoplanin yield and shorter fermentation period compared to those of S-45. We proposed that both selected recombinants had great industrial potentials.

### Analysis of deduced amino acid sequence in σ^HrdB^

**Figure 5:**
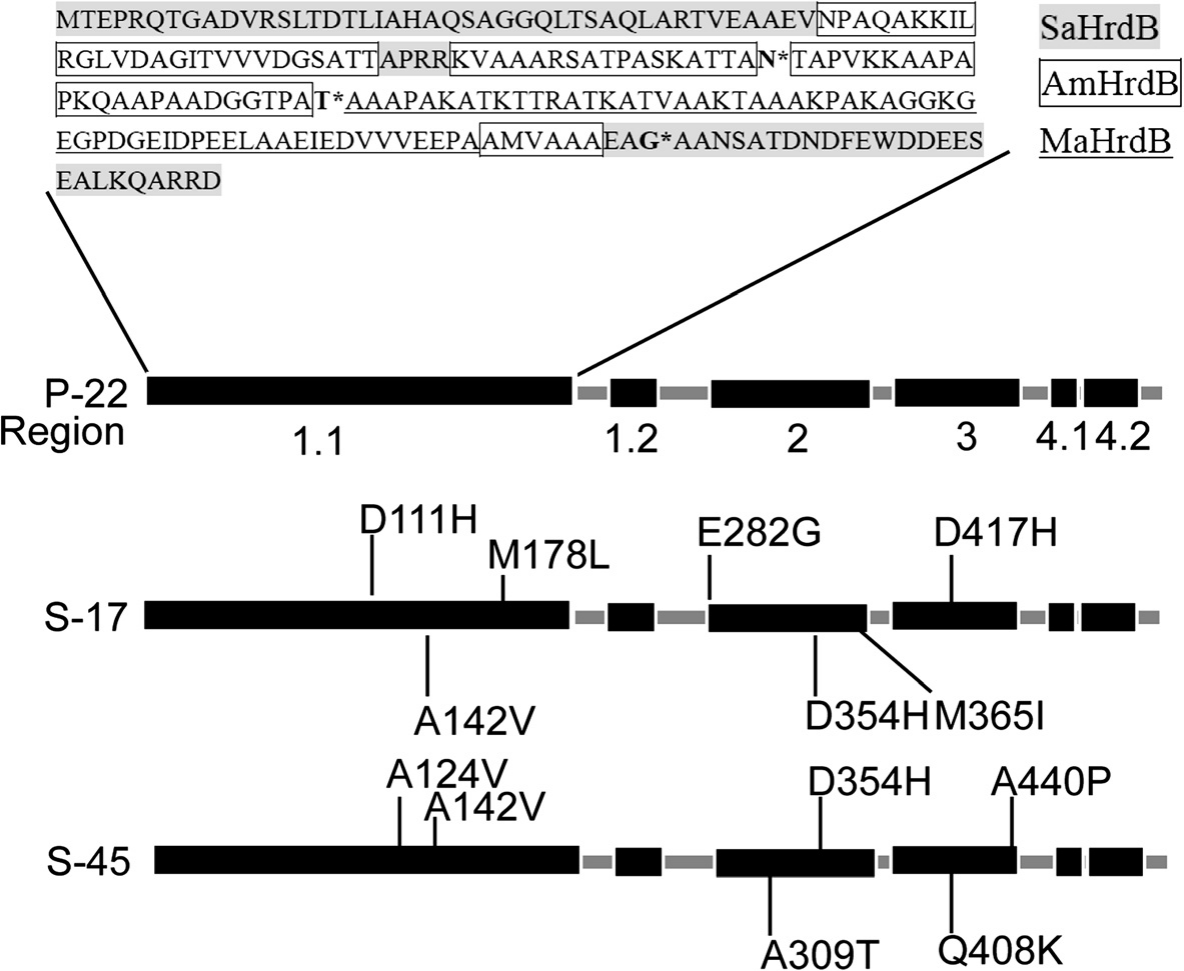
**Schematic mapping of the best chimera P-22 and mutation sites introduced by error-prone PCR in the isolated σ**^**HrdB **^**of the recombinants S-17 and S-45.** Conserved regions were shown in both the parent σ^HrdB^ and its evolved offspring. Triple parental contribution in region 1.1 of the hybrid was manifested (gray background, box and underline presented parts of σ^HrdB^ amino acid residues in *Salinispora arenicola* CNS-205, *Actinoplanes missouriensis* 431 and *Micromonospora aurantiaca* ATCC 27029 respectively), in which the asterisk labeled amino acids were obtained during the StEP process. Other regions of the chimera harbored the same amino acid residues as those in HrdB of *Actinoplanes missouriensis* 431.

Since the recombinants S-17 and S-45 presented high production elicited by the introduction of mutant *hrdB*, the gene sequence of desired mutant σ^HrdB^ should be analyzed. The shuffled mutant profile of σ^HrdB^ in P-22 was depicted by the sequence alignment with the three parental σ^HrdB^, as shown in Figure 5. It was found that DNA shuffling mainly occurred in region 1.1 of the chimera. Considering the evolved σ^HrdB^ of S-17 and S-45, site mutations elicited by error-prone PCR were scattered in 1.1, 2nd and 3rd regions. In the mutant σ^HrdB^ of S-17, there were seven novel mutations of D111T, A142V, M178L, E282G, D354H, M365I and D417H. For the mutant σ^HrdB^ of S-45, six mutations of A124V, A142V, A309T, D354H, Q408K and A440P were introduced. Apparently, A142V and D354H replacements existed in the two highest yield mutants, which indicated strongly this substitution a key role in DNA binding modulation. In addition, to evaluate the importance and necessity of these two mutation sites for high production, site-directed mutagenesis was performed in the shuffled *hrdB*, which amplified from P-22. Introducing a single point mutation A142V or D354H into the shuffled *hrdB* was found to make teicoplanin production in the host increase to 4.9 mg/ml or 4.6 mg/ml, respectively. The higher production (5.1 mg/ml) was achieved by simultaneously introducing the two mutations. It showed an obvious increase in yield compared to that of their parent P-22. The results suggested that these mutations were responsible for high yield in two best recombinants.

## Discussion

It is generally believed that fermentation phenotype is controlled by multiple genes existing in complicated cellular networks [9]. For a genome-annotated strain like *Streptomyces tsukubaensi*, it could be engineered to increase FK-506 production even by taking the whole metabolic network as a basis [24]. Transcriptional engineering can not only circumvent the trouble involved in completely deciphering the metabolic complexity, but also offer an efficient practical pathway to redirect the metabolic flux in the absence of metabolic network information. Because of the unique use of teicoplanin, an industrial strain of *A. teichomyceticus* has endured long-time industrial adaptation and several rounds of engineering. However, less knowledge of metabolic flux makes it difficult to generate higher yield recombinants. In this study, the industrial strain was successfully engineered and the yield-improved recombinants were obtained.

Artificial evolution of global transcription factors RpoD and SPT15 has been an attracted pathway to transcriptional engineering for phenotypic improvement in both *E. coli* and *Sacchoromryces cerevisiae*, respectively [9,10,25]. The corresponding global transcription factor σ^HrdB^ regulated morphogenesis and antibiotic production in *Steptomyces*[26]. The evolved σ^HrdB^ in *S. avermitilis* led to a remarkable increase in the production of avermectins [27]. However, no coding sequence of σ^HrdB^ in *A. teichomyceticus* was reported. Additionally, we also noticed that several exogenous transcription factors have been functionally expressed in new hosts by the way of either native or artificial form. For example, AvrBs3 from Gram-negative bacteria modulated transcription in the plant host [28]; Hac1 from filamentous fungi functioned in yeast [29]; and the most recently evolved IrrE from *Deinococcus radiodurans* was operated in *E. coli*[30]. However, dysfunctional heterologous expression is prevalent, and there’s no effective way to confirm whether one transcription factor works in a new host. Rational selection of exogenous transcription factor offers significant advantages over non-rational selection, avoiding obstacles towards general applicability of transcriptional engineering.

According to the molecular evolutionary analysis of σ^HrdB^ in several arbitrarily selected actinomycetes, three exogenous transcription factors were selected for mutation. Our analysis elicited that there were high similarity among principal sigma factors from the selected actinomycetes, with p-distance of 0.15-0.45 between groups. Why not *hrdB* from other genus of actinomycetes be selected? We proposed here the relatively reliable tactic. Actually, to select and shuffle other distant molecules can perhaps result in more rapid evolution. Therefore, mutational robustness should be considered in practical application when single exogenous one is selected.

With the native σ^HrdB^ of *A. teichomyceticus* L-27 keeping intact, recombinant regulators competed with it in binding the same cis-elements and interacting with the same components of the host L-27. In the present work, L-27 harboring any one of the three intact exogenous HrdB gave a significant lower yield (Figure 3). A combined strategy, rapid evolution through forming chimera followed by fine-tuned point modification, was adopted to engineer σ^HrdB^. Both shuffling and error-prone PCR generated the evolved σ^HrdB^ molecules leading to the increase of teicoplanin in *A. teichomyceticus* L-27. It was found that the shuffling process brought significant diversities in region 1.1 and generated a high-yield recombinant P-22. Not surprisingly, mutation was repressed due to perfect conservatism of other regions. Region 1.1 in the σ^HrdB^ affected the profile of self-inhibition [31], which was one explanation for the high productivity. Moreover, error-prone PCR made several point mutations in exogenous *hrdB* of P**-**22, and endowed the host with better performance. The mutation sites scattered in the region 1.1, 2 and 3. The region 2, the most conserved in σ^HrdB^, was responsible for -10 promoter recognition helix and the primary core RNA polymerase binding determinant [25]. As for the region 3, no functional profile was reported. In this study, site mutations were brought in region 3, but we did not investigate whether these mutations contributed to the yield improvement. No pressure was exerted during the screening progress, and parts of mutants showed the decreased yield in fermentation productivity test (Figure 3). S-17 with shortened fermentation period and high yield was identified in our program. A 120 h fermentation period of S-17 was comparable to that of *Actinoplanes teichomyceticus* ATCC31121 exhibiting higher fermentation temperature [12], which meant less cost from one batch culture in both energy conservation and temperature stability.

## Conclusion

It is difficult to further enhance yield of industrial strains resulting from long-time industrial adaptation and modification in several rounds. In this study, it was firstly illuminated that the σ^HrdB^ molecules shared strong similarity of amino acid sequences among some genera of actinomycetes. Combining this advantage with the ability of transcriptional engineering, exogenous sigma factor σ^HrdB^ was rationally selected and engineered to improve L-27. Several superior teicoplanin-producing strains with industrial potential were successfully constructed using less genetic operation and harmless mutagenic factors. It was the first time to investigate the issue on rational selection and engineering of exogenous transcription factor. Transcriptional engineering is a promising strategy to be investigated systematically. As its extension, our approach can be an alternative tool for efficient metabolic perturbation in more microorganisms, providing an ideal opportunity to analyze its fundamental principles and rapidly improve phenotypes in a host.

## Competing interests

The authors declare that they have no competing interests.

## Authors’ contributions

LY and HW designed and carried out most of the experiments. KW performed the high-throughput screening process. GL performed point mutation. LY and HW wrote this manuscript. All authors read and approved the final manuscript.
